# The health co-benefits and costs of climate adaptation interventions: A rapid scoping review and implications for policy and practice

**DOI:** 10.1016/j.joclim.2026.100666

**Published:** 2026-05-06

**Authors:** Annabelle Workman, Sophie Cullen, Hasini Gunasiri, Elise Moo, Vanora Mulvenna, Rohani Savage, Fran MacDonald, Kathryn J. Bowen

**Affiliations:** a Melbourne Climate Futures, The University of Melbourne, Grattan Street, Parkville 3010, Australia; b Melbourne School of Population and Global Health, Faculty of Medicine, Dentistry and Health Sciences, The University of Melbourne, Bouverie Street, Parkville 3010, Australia; c Office of the Chief Health Officer, Community and Public Health Division, Victorian Department of Health, Melbourne 3000, Australia; d Western Alliance for Greenhouse Action, Sunshine, 3020, Australia

**Keywords:** Climate change, Adaptation, Health co-benefits, Policy

## Abstract

**Background:**

Climate change is the biggest health threat of the 21st century, with health outcomes worsening across most climate-sensitive health parameters. The World Health Organization’s 2025 Global Action Plan on Climate Change and Health calls for health and climate issues to be better integrated in policy development as a matter of priority. Recent research indicates that climate adaptation interventions present an opportunity for substantial returns on investment when health and other outcomes are accounted for. This rapid scoping review explores a subset of the broader peer-reviewed literature assessing health co-benefits and costs of climate adaptation interventions.

**Methods:**

A search of PubMed, Scopus, and MEDLINE was carried out in August 2024 and 286 papers were screened for eligibility. 25 papers were deemed eligible for inclusion and data were extracted across multiple variables, including geographic location of study, reported cost of intervention, health outcomes and reported indicators.

**Results:**

Most of the 25 included papers were review articles and did not quantify or monetize health co-benefits. Only two of the 25 included papers reported the cost of featured climate adaptation interventions. A quarter of the included papers discussed potentially relevant indicators for reporting adaptation health co-benefits. Nature-based solutions and interventions relating to the built environment were the most common sectors covered.

**Conclusion:**

Recognizing the limitations of our findings given the subset of papers included in this scoping review, there appears to be an opportunity within the peer-reviewed literature to quantify the health co-benefits of climate adaptation interventions, report on their costs and benefits, and use standardized intervention typologies or measurement frameworks to assess effectiveness. Beyond the need for a more comprehensive and systematic review, future research must better evaluate the effectiveness and health outcomes of climate adaptation interventions and planning through appropriate study designs to enable evidence-based policy decisions that optimize the benefits of adaptation action.

## Highlights

• Limited quantification of health co-benefits of climate adaptation interventions in a subset of relevant papers.
• Very few studies in the eligible subset report costs or cost-benefit of climate adaptation measures.
• An opportunity to develop a standardized framework to measure the effectiveness of adaptation interventions.
• Greater intervention evaluation required to support policy and investment decisions.

## 1 Introduction

Climate change is recognized as the most catastrophic global health threat of our times [1]. Changes in climate are causing an increasing frequency and intensity of droughts, floods, fires, extreme heat and broader extreme weather events which pose direct and indirect risks and impacts to physical and mental health and wellbeing [2]. Indirect climate-related health risks include increased air pollution, food and water insecurity, changing patterns of disease, population migration, and destruction to human settlements [1].

Adverse health outcomes are worsening across most climate-related indicators. In 2022, for example, an additional 151 million people across 124 countries experienced heat- and drought-related food insecurity compared to 1981–2010 [1]. Further, an increase of 167 percent was recorded in heat-related deaths among people over 65 years in 2023 compared to the 1990s [1]. Effective adaptation action is therefore essential to protect populations from climate-related health risks and impacts.

In the context of climate action, "adaptation" refers to “the process of adjustment to actual or expected climate and its effects, in order to moderate harm or exploit beneficial opportunities” [3]. Common adaptation strategies include capacity-building and behavior change, policy and management reform, dedicated financing (via insurance schemes or contingency funds), early warning systems, and the development of climate-resilient and green infrastructure [4]. Adaptation often complements mitigation [5], which refers to strategies designed to “reduce emissions or enhance sinks of greenhouse gases” [3], such as transitioning to renewable energy or increasing the use of active or public transport.

The importance of pursuing improved health outcomes in climate policy development has been recognized at the highest levels of international decision-making. For example, in May 2025 at the 78th World Health Assembly, a Global Action Plan on Climate Change and Health was adopted [6]. The Plan calls on Member States to prioritize climate mitigation and adaptation policies that contribute to achieving health co-benefits. A health co-benefit is an additional positive effect that a policy or program, designed to respond to climate-related risks, has on individual or public health outcomes, thereby increasing the total benefit to society or the environment [3]. To date, there have been concerted efforts among the research community to characterize and quantify health co-benefits associated with mitigation measures [7,8], most notably relating to improved health outcomes from reduced exposure to fossil fuel-related air pollution in energy and transport sectors. This is largely a result of scientific advances in detection and attribution that enable the identification of causal pathways between exposure to climatic events and health outcomes [9].

However, the characterization and quantification of health co-benefits of adaptation interventions does not appear to have received the same level of attention as mitigation. In a 2021 scoping review of climate change and health research, the World Health Organization (WHO) found that only three of 2,181 included papers explicitly covered the health impacts of adaptation measures, with all relating to heat management [10]. Similarly, economic evaluations of adaptation interventions and their resulting health benefits historically have been absent from peer-reviewed literature. Average returns on adaptation investments can be substantial, with recent estimates concluding that over ten years, a USD$1 investment in adaptation provided more than USD$10 in benefits [5]. The largest gains were found to result from economic, social and environmental benefits rather than avoided losses. This suggests that adaptation interventions present an opportunity for substantial returns on investment when health and other outcomes are accounted for.

Globally, nations have failed to commit to or invest in domestic and international adaptation at a pace and scope commensurate with the proven health impacts and risks of climate change [11,12]. While all 59 national adaptation plans recently reviewed by WHO identify climate-sensitive health risks, these risks are rarely prioritized in terms of adaptation interventions [13]. Further, while some guidance on evaluation of such adaptation interventions is emerging [14], there is no consensus on measurement frameworks and indicators that might standardize data collection and support evidence-based decision-making. Poor access to data on the cost-effectiveness of adaptation interventions can act as a barrier to securing the critical investment needed to protect populations from climate-related health risks and impacts [[15], [16], [17]]. Since 2020, international adaptation financing to developing countries has decreased [18]. In 2023, adaptation interventions impacting health received less than a third of the Green Climate Fund’s adaptation funding [1].

In addition to insufficient evidence demonstrating the effectiveness of adaptation, other recognized barriers to delivering adaptation measures include limited intersectoral collaboration and the perceived difficulty of mobilizing social and political support [[19], [20], [21]]. Quantifying health co-benefits beyond avoided damages or losses could reveal the wide-ranging public health benefits of adaptation action, providing a powerful rationale for its prioritization in policy [22].

Accordingly, this rapid scoping review was undertaken on a subset of the broader peer-reviewed literature on climate-related health co-benefits to investigate an overarching research question: how are health co-benefits and/or costs (often referred to as co-harms or trade-offs) of climate adaptation interventions characterized?

## 2 Methods

We completed a rapid scoping review of peer-reviewed literature in August 2024 to assess the evidence on health co-benefits associated with climate adaptation interventions. Following PRISMA Extension for Scoping Reviews checklist [23], we undertook a search of PubMed, Scopus, and MEDLINE using a focused search strategy to obtain a specific subset of the broader climate-related health co-benefits peer-reviewed literature that contained the terms "climate," "co-benefit," "health," and "adaptation" (see Table S1 in the Supplementary Material). The intention of this scoping review was not to undertake a comprehensive and systematic review of the literature, but instead to identify peer-reviewed papers that explicitly drew connections between these key concepts. In total, 286 papers were initially identified and uploaded to Covidence.

### 2.1 Eligibility criteria

Articles were eligible for inclusion in the review if they were published in a peer-reviewed journal, had been published since 2010 in the English language, and discussed the health co-benefits of climate change adaptation interventions. Given adaptation interventions are often context-specific and place-based, where authors identified adaptation and health co-benefits and/or co-harms were explicitly referenced in relation to these interventions, articles were deemed eligible for inclusion. The scope was limited to peer-reviewed literature given time and resource constraints. Book chapters included in edited books were considered to have been peer-reviewed and were included.

**Fig. 1:**
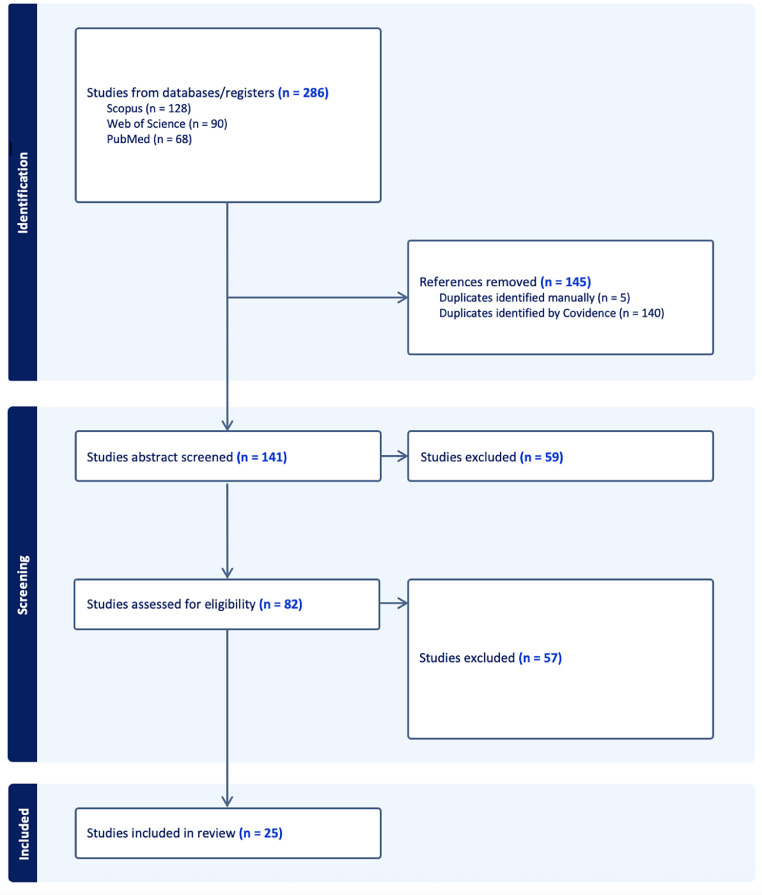
Summary of article selection process.

Following the automatic removal of 140 duplicates, one author (AW) completed abstract screening to determine eligibility. A total of 82 papers progressed to full-text screening and were then assessed for inclusion by two authors (AW, SC). Of these, 57 were deemed ineligible, with the primary reason for exclusion being no explicit reference to health co-benefits of adaptation interventions (many referenced mitigation interventions). 25 papers progressed to data extraction (see Fig. 1 below). 22 were published in peer-reviewed journals with the remaining 3 comprising chapters in edited books.

### 2.2 Data extraction

The 25 eligible articles were uploaded to an Excel data extraction template and were coded by one author (AW; see Table S2 in the Supplementary Material for the list of variables comprising the template). A second author (SC) completed an analysis of several papers selected at random to validate the data extraction process. Where needed, discrepancies in coding were discussed and a final categorization agreed. During the coding process, 19 additional articles were identified in the reference lists of eligible articles; however, all were deemed ineligible for inclusion as they made no explicit reference to health co-benefits of adaptation interventions.

## 3 Results

### 3.1 Study characteristics

Of the 25 included papers, approximately two-thirds (*n* = 18) were literature review articles, while the remaining (*n* = 7) involved the conduct of original research. Almost half (*n* = 12) of the eligible studies have been published since 2020 and most (*n* = 24) addressed measures or interventions that had been, or would be, implemented by governments. Most studies (*n* = 15) were global in context. Of note, very few (*n* = 2) reported the monetary cost of proposed or actual adaptation interventions. Adaptation measures relating to the built environment and/or nature-based solutions featured in most studies (*n* = 21). Almost all included studies (*n* = 24) implemented adaptation interventions targeting local communities. Approximately half (*n* = 12) of the included studies acknowledged their funding sources, with funders mostly based in Europe and the United States of America (USA). The remaining studies (*n* = 13) did not specify the funder or declared no funding source. Most (*n* = 17) studies focused solely on adaptation while the remaining studies (*n* = 8) considered both adaptation and mitigation measures concurrently. Table 1 presents an overview of study characteristics, while Table S2 in the Supplementary Material provides further detail on each included study.

**Table 1:** Summary of study characteristics, stratified by variable.

Variable		Count	References
Year of publication	2010 – 2015	5	[24], [25], [26], [27], [28]
	2016–2020	8	[29], [30], [31], [32], [33], [34], [35], [36]
	2021–2025	12	[37], [38], [39], [40], [41], [42], [43], [44], [45], [46], [47], [48]
Location of study	Global	15	[[24], [25], [26], [27], [28], [29],^31^,^34^,^35^,^37^,^38^,[41], [42], [43],^46^]
	USA	4	[30,32,40,44]
	China	2	[47], [48]
	Mexico	1	[33]
	India	1	[39]
	Australia	1	[36]
	Germany	1	[45]
Funding source	Europe/UK	5	[24], [27], [31], [37], [39], [45]
	USA	5	[26], [30], [32], [34], [44]
	Canada	1	[29]
	Not specified/ no funding received	13	[25,28,33,35,36,38,[40], [41], [42], [43],[46], [47], [48]]
Monetary cost of intervention reported	Yes	2	[33], [34], [35], [36], [37], [38], [39], [40], [41], [42], [43], [44]
	No	23	[24], [25], [26], [27], [28], [29], [30], [31], [32], [34], [35], [36], [37], [38], [39], [40], [41], [42], [43], [45], [46], [47], [48]
Adaptation system/ sector	Built environment	16	[25,26,29,30,32,34,[38], [39], [40], [41],[43], [44], [45], [46], [47], [48]]
	Nature-based solutions	16	[[24], [25], [26], [27],^29^,^31^,^34^,^35^,^38^,^39^,[41], [42], [43],[45], [46], [47]]
	Food systems	3	[37,38,43]
	Planning and design	5	[25,26,30,32,43]
	Emergency management	5	[25,28,36,46,48]
	Water	2	[33,34]
Subpopulation/ target group	Government/policymakers	1	[37]
	Community members (not specified)	17	[24,[26], [27], [28], [29], [30], [31],[33], [34], [35], [36],^39^,[41], [42], [43],^45^,^47^,^48^]
	Elderly	6	[25,28,30,32,38,46]
	Children	3	[32,38,40]
	People with low incomes	6	[30,32,38,40,44,46]
	People with chronic diseases	4	[25,28,32,46]
	People with poor quality or no housing	2	[32,38]
	Outdoor workers	1	[32]
	Marginalized/ socially disadvantaged groups	2	[25,32]
Response type	Considers adaptation interventions only	13	[25,[29], [30], [31], [32],^35^,^37^,^38^,^40^,^42^,[44], [45], [46]]
	Considers adaptation and mitigation interventions	12	[24,[26], [27], [28],^33^,^34^,^36^,^39^,^41^,^43^,^47^,^48^]
Health outcomes	Specified physical health co-benefits	20	[[24], [25], [26],^28^,[30], [31], [32], [33], [34], [35], [36], [37],^39^,^41^,[43], [44], [45], [46], [47], [48]]
	Specified mental health co-benefits	7	[24,27,[29], [30], [31],^35^,^46^]
	Health co-benefits mentioned but not specified	3	[38,40,42]
	Specified health co-harms	9	[[24], [25], [26], [27],^32^,^35^,^37^,^43^,^46^]
	Quantified health co-benefits	9	[28,31,33,39,[44], [45], [46], [47], [48]]
	Qualitative health co-benefits	16	[[24], [25], [25], [27],^29^,^30^,^32^,[34], [35], [36], [37], [38],[40], [41], [42], [43]]
Measurement framework and/or indicators identified	Yes	6	[29,36,40,45,47,48]
	No	19	[[24], [25], [26],[30], [31], [32], [33], [34],[37], [38], [39],[41], [42], [43], [44]]

### 3.2 Study findings

#### 3.2.1 Health outcomes

Health outcomes were either co-benefits or co-harms originating from adaptation actions. Six systems or sectors were selected to categorize climate adaptation actions, informed by the details of eligible studies, domestic as well as international adaptation reports: i) built environment; ii) nature-based solutions; iii) food systems; iv) planning and design; v) emergency management; and vi) water. Health co-benefits were categorized as either physical or mental for the purposes of the review. Articles were considered as ‘specifying’ a physical and/or mental health co-benefit if a health outcome was explicitly named. Three articles referred to health co-benefits but did not specify them [38,40,42]. Physical health co-benefits were specified in 20 of the 25 included articles, and comprised reductions in conditions such as cardiovascular, respiratory, infectious, water-borne and vector-borne diseases. Reductions in mortality and morbidity rates were also classified as specific physical health co-benefits. Many of the included articles mentioned health co-benefits related to heat (such as reduced heat-related illness and mortality or increased thermal comfort), often as the result of the cooling effects of nature-based solutions and green urban infrastructure. Seven of the 25 included articles specified mental health co-benefits of adaptation, such as reductions in depression or anxiety. Mental health co-benefits were frequently associated with nature-based solutions. For example, one of the included reviews [31] reported on a study that found that improvements in mental health were the “most notable” of the co-benefits associated with having 10 % more green space than average within one kilometer of one’s living environment, which reduced anxiety disorder and depression at decreased odds ratios of 0.95 and 0.96 respectively [49].

Nine of the 25 included articles named potential negative health impacts or health-related co-harms (*n* = 9) associated with adaptation measures. The health co-harms appearing most frequently pertained to increases in vector-borne disease, zoonotic disease, respiratory disease and allergies linked with greater exposure to wildlife, vectors and pollen-producing plants in expanded green spaces [[24], [25], [26],^32^,^35^,^43^,^46^].

#### 3.2.2 Adaptation interventions

Studies were categorized into the six adaptation systems or sectors based on the adaptation intervention(s) documented. Studies were classified in multiple sectors where more than one was discussed, and nearly two-thirds of included articles (*n* = 15) addressed multiple adaptation sectors.

The *built environment* sector was most prominent in eligible studies (*n* = 16). Passive building design (such as natural ventilation) and green urban infrastructure (such as green roofs) were linked with lower air temperatures and improved heat management, resulting in reduced heat-related illness or mortality [26,28,29,40,46]. Green urban design was also demonstrated to promote stormwater management and to reduce the risk and magnitude of urban flooding, producing health co-benefits such as reduced rates of water-borne disease, malaria, respiratory disease, psychological harm and flood-related injury [30,46].

In addition to their cooling effects, *nature-based solutions* (such as parklands, shaded refuges and other public green spaces) were also identified as increasing physical activity and thereby reducing diabetes, cardiovascular disease, respiratory disease and obesity [25,31,35,43,46]. Expansion of green space in China from 2011 to 2021 was estimated to have prevented 22, 893 deaths [47]. Greater proximity and access to green spaces were also associated with improved mental health [24,25,29,31,35], which was generally attributed to the increased opportunity for social connectivity and/or physical activity that these spaces afforded. In one review of green design studies, for example, it was reported that 40 percent canopy cover reduced stress levels and that views of greenery could increase attention span and cognitive performance [29].

Five of the included 25 studies addressed *emergency management*, generally finding that early warning systems reduced the number of deaths and hospital presentations from extreme heat and extreme weather events. One study linked heat warning systems to reduced heat-related illnesses, and air quality monitoring and alert systems to reduced respiratory illnesses like asthma [26]. Other emergency management measures included education [28], emergency shelters [28,48] and effective collaboration between communities, governments and non-government organizations [28]. One study found that adaptation strategies that targeted social capital, such as using buddy systems to check on neighbors and outreach initiatives for priority population groups, protected against heat-related illness during heat waves and improved overall health and wellbeing [25].

Changes in *food systems* were discussed in three studies in terms of enhanced urban agrobiodiversity, improved water management, and improved land use. They were associated with reductions in cardiovascular disease risk [37], heat-related illness and vector-borne disease [38], as well as with improved nutrition [37,43]. Only two articles from our search discussed *water* interventions such as mangrove restoration, wastewater use, piped drinking water and improved sewerage systems, and their capacity to reduce enteric diseases, dengue, and chikungunya [33,34]. Regarding *planning and design*, adaptation measures described included improved road design, pedestrian- and bicycle-friendly infrastructure and improved public transport, which were linked with increased physical activity and thereby potential reductions in obesity and cardiovascular disease [25].

#### 3.2.3 Quantification of health co-benefits

Articles were considered as quantifying health co-benefits if they provided a numerical measure of a health outcome associated with a specific adaptation intervention. Many of the 25 included articles (*n* = 16) did not quantify health co-benefits but referred to them qualitatively. Articles sometimes assumed or implied health outcomes by referring to minimized risk or exposure, but these were not considered as specifying co-benefits for coding purposes. For example, one study [43] linked adaptive urban planning (in this case, risk zoning and infrastructure relocation) to changed risks (in this case, to flooding and fire) or improved adaptive capacities but did not explicitly specify health-related outcomes.

Of the included studies reporting original research (*n* = 7), only one generated new data on health outcomes from the implementation of an adaptation measure, i.e. a retrospective study [45]. The authors conducted biophysical assessments and surveys to quantify the effect of urban green structure on thermal comfort in four courtyards in Potsdam, Germany. The remaining six original research studies tended to create models of anticipated outcomes for prospective adaptation measures, or to apply existing, generalized measures to propose expected outcomes in a specific context. For example, one eligible study cited WHO estimations that piped drinking water reduced diarrheal disease by 28 percent to anticipate similar co-benefits for a potential water sanitation project in Morelos state, Mexico [33].

#### 3.2.4 Research gaps

Acknowledging that our rapid scoping review analyzed only a subset of the broader co-benefits literature, our results highlighted three gaps that warrant further exploration. Firstly, only two of the 25 included studies captured in our focused search reported the monetary costs of the adaptation measures they described. Both compared the projected health co-benefits produced by tiered investment scenarios. The first used modelling to compare three potential levels of energy efficiency in public housing in Phoenix (Levels 2 and 3 costing an additional USD$4336 and USD$36,522 respectively, compared to Level 1) [44]. The authors found that accounting for climate-related exposures, such as a reduction in indoor air temperature, reduced the payback periods for the investments by up to six years and potentially avoided health costs related to hospitalizations, premature mortality, asthma and respiratory conditions. The second compared a sanitation intervention that considered an adaptation intervention (USD$9.25 million) alongside an adaptation and mitigation intervention (USD$35.63 million) with baseline and business-as-usual scenarios in Morelos, Mexico [33]. The authors estimated that reductions in dengue from improved sanitation would save a minimum of USD$5750 per medical attention per dengue case. Another eligible study [46] included in its review an article that found that minimizing the quantity of people with sick building symptoms through increased ventilation could reduce healthcare costs by up to USD$11.5 million, while also noting a potential increase of up to USD$207 million in health costs arising from trade-offs, such as exposure to ozone and particulate matter. While some included studies reported the economic losses from climate-related impacts, suggested cost-benefit ratios, or qualitatively described how adaptation might enhance labor productivity and reduce healthcare costs, these three studies were the only to monetize adaptation investments and their health co-benefits.

Secondly, only a quarter (*n* = 6) of the 25 eligible studies used or proposed measurement frameworks or indicators to measure the health co-benefits of climate adaptation interventions [29,36,40,45,47,48]. One study introduced a typology of urban green space adaptations and six categories of health metrics, including disease recovery, physical activity and mental health [29]. Other studies drew from existing frameworks, including the UN Sustainable Development Goals [40], and spatial indicators from an existing national standard in China [48]. Thirdly, most studies were published in global (*n* = 15) or high-income country *(n* = 6) contexts, with only four of the 25 included studies identified from low and middle-income countries. This initial finding from our focused search suggests a potential gap in evidence on the health co-benefits of climate adaptation interventions in lower-resource settings which requires further investigation through a more comprehensive and systematic review.

## 4 Discussion

This rapid scoping review used a focused search strategy to identify studies explicitly linking the concepts of climate, health, co-benefits and adaptation. It synthesized evidence on the health co-benefits of climate adaptation interventions implemented across six sectors, including characterization of health outcomes and efforts to quantify intervention impact from 25 eligible studies. Most articles qualitatively described health co-benefits without reporting empirical data, and several studies referenced reduced risk of exposure to hazards, but not the resulting health outcomes. Built environment and nature-based solutions were the sectors most frequently linked to health co-benefits, appearing in a total of 22 studies. By comparison, emergency management was covered in only five of the included studies, despite indications that such interventions can effectively reduce heat- and extreme weather-related morbidity and mortality [28,48].

Further, two of the 25 included studies reported the monetary costs of the adaptation measures they featured, and very few monetized the health benefits associated with the implementation of adaptation interventions. There was also limited engagement with measurement frameworks and the use of commonly used health or other indicators among the studies captured in our focused search. These are critical components of monitoring and evaluation for the purposes of establishing intervention efficacy, their absence here indicating the need for a more comprehensive review. As with broader climate change and health literature, there is a paucity of peer-reviewed evidence on health co-benefits of adaptation from low and middle-income country contexts [50,51], where populations face disproportionate risks from inadequate climate adaptation.

Of the 25 studies included in our rapid scoping review, there was no standardized or agreed typology of adaptation sector or system, making comparison of interventions and recommendations across jurisdictions challenging. Second, the limited consideration of health co-benefits associated with emergency management interventions presents an under-researched area where there is an opportunity to expand the existing literature. Similarly, this rapid scoping review would suggest that there is a need for further guidance on measurement frameworks and indicators to support comparable evaluations of adaptation measures.

Acknowledging the need to be cautious in interpreting the generalizability of our findings, our results have potential implications for policy and practice. In particular, the limited consideration of the monetary cost of the intervention in conjunction with limited quantification and monetization of associated health co-benefits will likely create significant barriers for policymakers who are often required to establish cost-effectiveness to justify investment in proposed adaptation measures. Further, while the quantification of health co-benefits associated with adaptation interventions is unsurprising given known attribution difficulties, it nevertheless is an area of identified need for policymakers so that they may better understand the causal pathways between interventions and outcomes. Finally, appropriate monitoring and evaluation of interventions and their impacts on health outcomes can also enable the justification of adaptation intervention investments [52], and as such, deserve greater attention.

Our results also have implications for future research. First, our findings establish the imperative for a more comprehensive review of the literature on the health co-benefits and costs of adaptation interventions, to determine if our results involving a subset of the literature are representative and applicable of the broader literature on climate-related health co-benefits of adaptation interventions. If our results are broadly reflective of the literature, then there is a need to better characterize, and ideally quantify, health outcomes associated with reduced risk of exposure to hazards. Moreover, while prospective modelling studies can be useful, there is also a clear need to invest further in retrospective and iterative studies that monitor and evaluate the health impacts of climate adaptation interventions. Second, further investigation into the effectiveness of adaptation planning, such as the implementation of early warning systems or the integration of adaptation into government policy, could improve the reach and impact of such measures. Third, while many of our included studies were global in focus, a minority reported on interventions implemented in developing contexts. Investment in research undertaken in developing settings where climate adaptation needs are most acute is essential, particularly to assist these countries with accessing climate adaptation finance. Additionally, the main funders of our included studies were from Europe and the USA. In a dynamic research funding landscape, additional funders will need to be identified to support future studies.

The findings from this rapid scoping review informed the development of an accessible, evidence-based decision support tool designed to enable climate policymakers working across sectors to account for health considerations in the development of adaptation policy, planning and investment [53]. This process was modelled on the successful design of an equivalent mitigation decision support tool [54].

### 4.1 Study limitations

Firstly, as previously mentioned, we acknowledge that this rapid scoping review is far from exhaustive as the search was predicated on the identification of measures or strategies resulting in explicitly articulated health co-benefits and/or costs that have been implemented for climate adaptation purposes. A wide range of studies exist that consider the health impacts of, for example, green space, without acknowledging that such interventions are motivated by climate change or are considered adaptation interventions. The use of general and limited search terms was designed to investigate literature that explicitly linked health impacts to climate change and co-benefits to adaptation actions, but is insufficiently extensive to capture all potentially relevant studies, including those that refer to specific impacts or hazards without reference to more general terms, such as ‘climate’ and ‘health’. Further, this review was restricted to peer-reviewed literature, potentially overlooking a rich body of evidence available from non-peer-reviewed sources and introducing possible publication bias in the sample. Accordingly, to validate the findings of our focused review, there is an opportunity to expand the search strategy and inclusion criteria used in this study to investigate evidence from other sources, such as evaluations and reports from local governments, community-led projects, or non-government organizations.

Despite the narrow parameters of our review, the eligible articles included provide a foundational understanding of the discourse around the health co-benefits concept and climate adaptation interventions from which further research may draw.

## 5 Conclusion

The threat to population health posed by climate change calls for immediate and meaningful action to reduce health risks and minimize impacts. This rapid scoping review of a subset of the peer-reviewed literature on the health co-benefits and costs of climate adaptation interventions found that most of the 25 eligible studies documented health co-benefits qualitatively, focused on physical health outcomes and did not reference relevant measurement frameworks or indicators. The review identifies an opportunity to further investigate the broader literature to strengthen the evidence on the effectiveness of climate adaptation interventions to improve health outcomes. It demonstrates the need to support policy development by prioritizing further research that i) quantifies the health co-benefits associated with adaptation interventions as well as reporting these qualitatively, ii) proposes standardized measurement frameworks to support the monitoring and evaluation of adaptation interventions, iii) reports on monetary costs and cost-benefit analysis, and iv) addresses adaptation sectors and contexts currently underrepresented in the literature, such as emergency management systems and developing country contexts.

## Funding

This study was funded by a Victorian Health Promotion Foundation (VicHealth) Impact ResearchGrant.

## CRediT authorship contribution statement

**Annabelle Workman:** Writing – original draft, Methodology, Funding acquisition, Formal analysis, Data curation, Conceptualization. **Sophie Cullen:** Writing – original draft, Formal analysis, Data curation. **Hasini Gunasiri:** Writing – review & editing. **Elise Moo:** Writing – review & editing, Project administration, Methodology, Conceptualization. **Vanora Mulvenna:** Writing – review & editing, Conceptualization. **Rohani Savage:** Writing – review & editing, Conceptualization. **Fran MacDonald:** Writing – review & editing, Methodology, Funding acquisition, Conceptualization. **Kathryn J. Bowen:** Writing – review & editing, Methodology, Funding acquisition, Conceptualization.

## Declaration of competing interest

The authors declare that they have no known competing financial interests or personal relationships that could have appeared to influence the work reported in this paper.

## Appendix Supplementary materials

Image, application 1

## Footnotes

Supplementary material associated with this article can be found, in the online version, at doi:10.1016/j.joclim.2026.100666.
