# Supplementary material for: The health co-benefits and costs of climate adaptation interventions: A rapid scoping review and implications for policy and practice
Source: J Clim Chang Health. 2026 May 6;29:100666. doi: 10.1016/j.joclim.2026.100666 (PMC13158384; doi:10.1016/j.joclim.2026.100666)
Supplement: Supplementary file 1 [file mmc1.docx]

**Supplementary Material**

Table S1. Search strategy to identify eligible papers for the rapid scoping review on health co-benefits of climate adaptation interventions

| **Date of search** | **Database** | **Search terms** | **Search results** |
| --- | --- | --- | --- |
| 6 August 2024  Limited to date range of 2010-2024 and published in English | PubMed | climate [Title/Abstract] AND co-benefit* [Title/Abstract] AND health [Title/Abstract] AND adaptation [Title/Abstract] | 68 |
|  | MEDLINE | AB climate AND AB health AND AB co-benefit* AND AB adaptation | 89 |
|  | Scopus | AB climate AND AB health AND AB co-benefit* AND AB adaptation | 127 |

Table S2. Overview of 25 eligible studies included in the rapid scoping review on health co-benefits of climate adaptation interventions

| **First author and year** | **Location of study** | **Type of study** | **Subpopulation(s)/ Target group(s)** | **Funding body (if reported)** | **Costs (if reported)** | **Policy response;**  **Sector(s)** | **Health outcome(s)** | **Measurement/ indicator(s)** |
| --- | --- | --- | --- | --- | --- | --- | --- | --- |
| Crona et al. (2023) [1] | Global | Literature review | Government | Europe | Not reported | Adaptation;  Food systems | **Co-benefits**  Physical: Reduction in cardiovascular risk and food-sensitive deficiencies from increased uptake of ‘blue foods’  Mental: No mention  *Qualitative*  **Co-harms**  Food insecurity due to increased uptake of ‘blue foods’ | None identified |
| Demuzere et al. (2014)  [2] | Global | Literature review | General community (not specified) | Europe | Not reported | Adaptation and mitigation;  Nature-based solutions | **Co-benefits**  Physical: Range of physical co-benefits from green urban infrastructure, such as improved thermal comfort and reduced likelihood of obesity from increased physical activity  Mental: lower rates of anxiety and depression from proximity to green space  *Qualitative*  **Co-harms**  Range of trade-offs, such as over-cooling from tree coverage in colder climates, and vector-borne and zoonotic disease from increase in animals/insects | None identified |
| Sheehan (2023) [3] | Global | Original research | Elderly; children; people with low incomes; people living in poor quality housing | Not specified | Not reported | Adaptation;  Built environment, food systems, nature-based solutions | **Co-benefits**  Physical: Reductions in heat-, vector-, and air pollution-related illness from urban agrobiodiversity and adaptation planning  Mental: No mention  *Qualitative*  **Co-harms**  No mention | None identified |
| Cheng et al. (2013) [4] | Global | Literature review | Seniors; people with chronic illness; socially disadvantaged people | Not specified | Not reported (but recognition that limited information is available) | Adaptation;  Built environment, nature-based solutions, transport, emergency management | **Co-benefits**  Physical: Range of physical co-benefits, such as reduced heat-related illness, obesity, and cardiovascular disease  Mental: Unspecified improvements to mental health  *Qualitative*  **Co-harms**  Spread of health misinformation from social capital strategies; increased allergic disease and pests from urban design strategies | None identified |
| Limaye et al. (2023) [5] | India | Original research | Community members (not specified) | Europe | Not reported | Adaptation and mitigation; Built environment, nature-based solutions | **Co-benefits**  Physical: Fewer annual all-cause and non-accidental deaths from reduced air pollution  Mental: No mention  *Quantitative*  **Co-harms**  No mention | None identified |
| Barron et al. (2019) [6] | Global | Literature review | Community members (not specified) | Canada | Not reported | Adaptation;  Built environment, nature-based solutions | **Co-benefits**  Physical: Unspecified health outcomes from increased physical activity and protection from extreme heat; thermal comfort  Mental: Increased attention span and cognitive performance, reduced stress, improved mental health from viewing and interacting with green spaces  *Qualitative*  **Co-harms**  No mention; notes ‘gentrification’ as trade-off | Health indicators included in Appendix |
| Houghton et al. (2017)  [7] | USA | Literature review | Elderly; low income; vulnerable populations | USA | Not reported | Adaptation;  Built environment, Planning and design | **Co-benefits**  Physical: Range of physical co-benefits from reduced exposure to flood risk, such as reduced infectious, water-borne and vector-borne disease and reduced risk of injury and mortality  Mental: Reduced psychological harm for survivors from facility design and reduced stress during evacuation events from transport management  *Qualitative*  **Co-harms**  No mention | Not identified |
| Chiabai et al. (2018)  [8] | Global | Literature review | Community members (not specified) | Europe | Not reported | Adaptation;  Nature-based solutions | **Co-benefits**  Physical: Range of physical co-benefits, such as reduced blood pressure, obesity and diabetes from increased physical activity. Note higher birthweight and reduced stroke mortality.  Mental: Reduced stress, depression and anxiety  *Quantitative*  **Co-harms**  No mention | Not identified |
| Houghton et al. (2019)  [9] | USA | Literature review | Children; the elderly; chronic disease sufferers; people living in poverty; non-Hispanic Black people; homeless populations; outdoor workers | USA | Not reported | Adaptation;  Built environment, Planning and design | **Co-benefits**  Physical: Range of physical co-benefits; emphasis on reduced heat-related stress, morbidity and mortality from minimizing sprawl and urban heat island effect  Mental: Unspecified improvements to mental health and wellbeing from protecting biodiversity  *Qualitative*  **Co-harms**  Increased risk of respiratory disease from increase in pollen-producing plants | Not identified |
| Harlan et al. (2011) [10] | Global | Literature review | Community members (not specified) | USA | Not reported | Adaptation and mitigation;  Built environment, nature-based solutions, **Planning and design** | **Co-benefits**  Physical: Range of physical co-benefits, such as reduced heat-related deaths and illnesses from warning systems, green roofs and urban forests  Mental: No mention  *Qualitative*  **Co-harms**  Increase in allergies from urban forest; acknowledge general trade-offs apparent in planning phase | Not identified |
| Houghton (2023) [11] | USA | Original research | People with low incomes; children | Not specified | Not reported | Adaptation;  Built environment | **Co-benefits**  Physical: Unspecified health outcomes related to heat, air pollution and bike/pedestrian safety  Mental: No mention  *Qualitative*  **Co-harms**  Unspecified; acknowledge that co-impacts can also be negative | Community metrics and UN SDGs associated with each intervention |
| Luyten et al. (2023) [12] | Global | Literature review | Community members (not specified) | Not specified | Not reported | Adaptation and mitigation;  Built environment, nature-based solutions | **Co-benefits**  Physical: Range of physical co-benefits, such as reduced non-communicable diseases  Mental: No mention (they note under-representation of mental health co-benefits)  *Qualitative*  **Co-harms**  Unspecified negative health impacts reported in their included articles | Not identified |
| Aghaloo et al. (2024)  [13] | Global | Literature review | Community members (not specified) | Not specified | Not reported | Adaptation;  Nature-based solutions | **Co-benefits**  Unspecified health outcomes associated with nature-based solutions  *Qualitative*  **Co-harms**  No mention | Not identified |
| Sharifi (2022) [14] | Global | Literature review | Community members (not specified) | Not specified | Not reported | Adaptation and mitigation;  Food systems, built environment, transport, nature-based solutions | **Co-benefits**  Physical: Range of physical co-benefits, such as reduced obesity, diabetes and cardiovascular disease from increased physical activity and reduced malnutrition from urban agriculture  Mental: Unspecified mental health outcomes from physical activity and active transportation  *Qualitative*  **Co-harms**  Reduced satisfaction and mental health from restricted vehicle usage, spread of infectious and vector-borne diseases and allergies from increased wildlife | Not identified |
| Baniassadi et al. (2021) [15] | USA | Original research | People with low incomes | USA | Energy efficiency investments across two scenarios | Adaptation;  Built environment | **Co-benefits**  Physical: Reduced heat-related hospitalization and mortality risks; reduced spread of indoor airborne diseases from increased air circulation  Mental: No mention  *Quantitative*  **Co-harms**  No mention | Not identified |
| Gonzalez at al. (2019)  [16] | Mexico | Original research | Community members (not specified) | Not specified | Sanitation and climate investments across two scenarios | Adaptation and mitigation;  Water | **Co-benefits**  Physical: Reduced water-borne diseases from access to clean water, reduced dengue and chikungunya from improved sanitation  Mental: No mention  *Quantitative*  **Co-harms**  No mention | Not identified |
| Spencer et al. (2016)  [17] | Global | Original research | Community members (not specified) | USA | Not reported | Adaptation and mitigation;  Nature-based solutions, built environment, water | **Co-benefits**  Physical: Reduced vector-borne disease from protecting forest ecosystems, reduced flood-related deaths and injuries from restoring mangrove systems, decreased fecal-oral disease transmission and increased food security from improved sanitation  Mental: Unspecified mental health outcomes from public green space and intact forest ecosystems  *Qualitative*  **Co-harms**  No mention; note in introduction about trade-off between adaptation and mitigation | Not identified |
| Schmidt et al. (2021)  [18] | Germany | Original research | Community members (not specified) | Europe | Not reported | Adaptation;  Built environment, nature-based solutions | **Co-benefits**  Physical: Improved thermal comfort  Mental: No mention; mention impact on social engagement but not associated mental health outcomes  *Quantitative*  **Co-harms**  Unspecified potential trade-offs of close interaction between people and urban nature | Measurement framework for establishing link between intervention and health outcomes |
| Sharifi et al. (2021) [19] | Global | Literature review | Elderly; people with low incomes; people with chronic conditions | Not specified | Not reported | Adaptation;  Built environment, nature-based solutions, emergency management | **Co-benefits**  Physical: Range of physical co-benefits, such as reduced heat-related stress and mortality and reduced cardiovascular disease from urban greenery and nature-based solutions  Mental: Alleviated anxiety from tree canopy exposure, slower cognitive decline from proximity to nature, stress reduction  *Quantitative*  **Co-harms**  Spread of vector-borne disease, zoonotic disease and pollen allergies from nature-based solutions; sick building syndrome | Not identified |
| Braubach et al. (2017)  [20] | Global | Literature review | Community members (not specified) | Not specified | Not reported | Adaptation;  Nature-based solutions | **Co-benefits**  Physical: Reduced allergy risk and improved immune system from exposure to biodiversity; reduced obesity, cardiovascular risk, cancer and osteoporosis from increased physical activity in green space  Mental: Cognitive benefits and alleviated stress and depression from contact with nature  *Qualitative*  **Co-harms**  Increased risk of injury, allergies, vector-borne disease and infectious disease | Not identified |
| Thomas et al. (2014)  [21] | Global | Literature review | Community members (not specified) | Europe | Not reported | Adaptation and mitigation;  Nature-based solutions | **Co-benefits**  Physical: Unspecified physical health outcomes  Mental: Increased self-reported wellbeing from green spaces  *Qualitative*  **Co-harms**  Reduced sense of freedom, independence and security from reduced car usage | Not identified |
| Smith et al. (2014) [22] | Global | Literature review | Elderly; people with chronic conditions | Not specified | Not reported | Adaptation and mitigation;  Emergency management | **Co-benefits**  Physical: Reduced cyclone-related mortality from planning, reduced mortality from heat wave early warning systems, reduced heat-related medical conditions from green space  Mental: Unspecified mental health outcomes of urban green space  *Quantitative*  **Co-harms**  No mention | Not identified |
| Cai et al. (2022)  [23] | China | Literature review | Community members (not specified) | Not specified | Not reported | Adaptation and mitigation;  Built environment, nature-based solutions | **Co-benefits**  Physical: Reduced mortality due to green space and air conditioning  Mental: No mention  *Quantitative*  **Co-harms**  No mention | Health indicators included |
| Ji et al. (2023) [24] | China | Literature review | Community members (not specified) | Not specified | Not reported | Adaptation and mitigation;  Built environment, emergency management | **Co-benefits**  Physical: Reduced mortality due to heatwave early warning system, air conditioning and urban planning; range of physical co-benefits from green space  Mental: No mention  *Quantitative*  **Co-harms**  No mention; acknowledges potential mitigation trade-offs with air conditioning use increased CO_2_ emissions | Reference to spatial indicators |
| Zhang et al. (2018) [25] | Australia | Literature review | Community members (not specified) | Not specified | Not reported | Adaptation and mitigation; Emergency management | **Co-benefits**  Physical: Reduced ambulance calls, emergency department presentations and hospitalizations from heatwave early warning  Mental: No mention  *Qualitative*  **Co-harms**  No mention | Health indicators included |

**References**

1. Crona BI, Wassenius E, Jonell M, Koehn JZ, Short R, Tigchelaar M, et al. Four ways blue foods can help achieve food system ambitions across nations. Nature. 2023;616(7955):104-12. https://doi.org/10.1038/s41586-023-05737-x

2. Demuzere M, Orru K, Heidrich O, Olazabal E, Geneletti D, Orru H, et al. Mitigating and adapting to climate change: multi-functional and multi-scale assessment of green urban infrastructure. J Environ Manage. 2014; 146:107-15. https://doi.org/10.1016/j.jenvman.2014.07.025

3. Sheehan MC. Urban agrobiodiversity, health and city climate adaptation plans. Bull World Health Organ. 2023;101(2):121-9. https://doi.org/10.2471/BLT.22.288857

4. Cheng JJ, Berry P. Health co-benefits and risks of public health adaptation strategies to climate change: a review of current literature. Int J Public Health. 2013;58(2):305-11. https://doi.org/10.1007/s00038-012-0422-5

5. Limaye VS, Magal A, Joshi J, Maji S, Dutta P, Rajput P, et al. Air quality and health co-benefits of climate change mitigation and adaptation actions by 2030: an interdisciplinary modeling study in Ahmedabad, India. Environ Res Health. 2023;1(2):021003. https://doi.org/10.1088/2752-5309/aca7d8

6. Barron S, Nitoslawski S, Wolf KL, Woo A, Desautels E, Sheppard SRJ. Greening Blocks: A Conceptual Typology of Practical Design Interventions to Integrate Health and Climate Resilience Co-Benefits. Int J Environ Res Public Health. 2019;16(21). https://doi.org/10.3390/ijerph16214241

7. Houghton A, Castillo-Salgado C. Health Co-Benefits of Green Building Design Strategies and Community Resilience to Urban Flooding: A Systematic Review of the Evidence. Int J Environ Res Public Health. 2017;14(12). https://doi.org/10.3390/ijerph14121519

8. Chiabai A, Quiroga S, Martinez-Juarez P, Higgins S, Taylor T. The nexus between climate change, ecosystem services and human health: Towards a conceptual framework. Sci Total Environ. 2018; 635:1191-204. https://doi.org/10.1016/j.scitotenv.2018.03.323

9. Houghton A, Castillo-Salgado C. Associations between Green Building Design Strategies and Community Health Resilience to Extreme Heat Events: A Systematic Review of the Evidence. Int J Environ Res Public Health. 2019;16(4). https://doi.org/10.3390/ijerph16040663

10. Harlan SL, Ruddell DM. Climate change and health in cities: impacts of heat and air pollution and potential co-benefits from mitigation and adaptation. Current Opinion in Environmental Sustainability. 2011;3(3):126-34. https://doi.org/10.1016/j.cosust.2011.01.001

11. Houghton A. Co-benefit Design: A Method for Catalyzing Progress on Global Climate and Sustainable Development Goals by Raising Adaptation and Population Health to the Same Level of Importance as Mitigation in the Design Process. In: Faircloth B, Pedersen Zari M, Ramsgaard Thomsen M, Tamke M, editors. Design for Climate Adaptation Proceedings of the UIA World Congress of Architects Copenhagen 2023. Cham, Switzerland: Springer Nature Switzerland; 2023. p. 199-208.

12. Luyten A, Winkler MS, Ammann P, Dietler D. Health impact studies of climate change adaptation and mitigation measures – A scoping review. The Journal of Climate Change and Health. 2023;9. https://doi.org/10.1016/j.joclim.2022.100186

13. Aghaloo K, Sharifi A, Habibzadeh N, Ali T, Chiu Y-R. How nature-based solutions can enhance urban resilience to flooding and climate change and provide other co-benefits: A systematic review and taxonomy. Urban Forestry & Urban Greening. 2024;95. https://doi.org/10.1016/j.ufug.2024.128320

14. Sharifi A. Sustainability and Resilience Co-benefits and Trade-Offs of Urban Climate Change Adaptation and Mitigation Measures. In: Lackner M, Sajjadi B, Chen W-Y, editors. Handbook of Climate Change Mitigation and Adaptation 3rd ed. Cham, Switzerland: Springer Nature Switzerland; 2022. p. 1369-404.

15. Baniassadi A, Heusinger J, Gonzalez PI, Weber S, Samuelson HW. Co-benefits of energy efficiency in residential buildings. Energy. 2022;238. https://doi.org/10.1016/j.energy.2021.121768

16. González IN, Cisneros BJ, Hernández NA, Rojas RM. Adaptation and mitigation synergies to improve sanitation: a case study in Morelos, Mexico. Journal of Water and Climate Change. 2019;10(3):671-86. https://doi.org/10.2166/wcc.2018.121

17. Spencer B, Lawler J, Lowe C, Thompson L, Hinckley T, Kim S-H, et al. Case studies in co-benefits approaches to climate change mitigation and adaptation. Journal of Environmental Planning and Management. 2016;60(4):647-67. https://doi.org/10.1080/09640568.2016.1168287

18. Schmidt K, Walz A. Ecosystem-based adaptation to climate change through residential urban green structures: co-benefits to thermal comfort, biodiversity, carbon storage and social interaction. One Ecosystem. 2021;6. https://doi.org/10.3897/oneeco.6.e65706

19. Sharifi A, Pathak M, Joshi C, He B-J. A systematic review of the health co-benefits of urban climate change adaptation. Sustainable Cities and Society. 2021;74. https://doi.org/10.1016/j.scs.2021.103190

20. Braubach M, Egorov A, Mudu P, Wolf T, Thomspon C, Martuzzi M. Effects of Urban Green Space on Environmental Health, Equity and Resilience. In: Kabisch N, Korn H, Stadler J, Bonn A, editors. Nature-based solutions to Climate Change Adaptation in Urban Areas Linkages between Science, Policy and Practice. Cham, Switzerland: Springer Nature; 2017. p. 187-206.

21. Thomas F, Sabel CE, Morton K, Hiscock R, Depledge MH. Extended impacts of climate change on health and wellbeing. Environmental Science & Policy. 2014; 44:271-8. https://doi.org/10.1016/j.envsci.2014.08.011

22. Smith KR, Woodward A, Campbell-Lendrum D, Chadee DD, Honda Y, Liu Q, et al. Human health: impacts, adaptation, and co-benefits. In: Field CB, Barros VR, Dokken DJ, Mach KJ, Mastrandrea MD, Bilir TE, et al., editors. Climate Change 2014: Impacts, Adaptation, and Vulnerability Part A: Global and Sectoral Aspects Contribution of Working Group II to the Fifth Assessment Report of the Intergovernmental Panel on Climate Change. Cambridge, UK and New York, USA: Cambridge University Press; 2014. p. 709-54.

23. Cai W, Zhang C, Zhang S, Bai Y, Callaghan M, Chang N, et al. The 2022 China report of the Lancet Countdown on health and climate change: leveraging climate actions for healthy ageing. Lancet Public Health. 2022;7(12):e1073-e90. https://doi.org/10.1016/S2468-2667(22)00224-9

24. Ji JS, Xia Y, Liu L, Zhou W, Chen R, Dong G, et al. China's public health initiatives for climate change adaptation. Lancet Reg Health West Pac. 2023; 40:100965. https://doi.org/10.1016/j.lanwpc.2023.100965

25. Zhang Y, Beggs PJ, Bambrick H, Berry HL, Linnenluecke MK, Trueck S, et al. The MJA-Lancet Countdown on health and climate change: Australian policy inaction threatens lives. Med J Aust. 2018;209(11):474. https://doi.org/10.5694/mja18.00789
